# Stage-Specific Prognostic Impact of Longitudinal Body Composition Changes in Patients with Pancreatic Ductal Adenocarcinoma Treated with FOLFIRINOX: A Dual-Cohort Study

**DOI:** 10.3390/jcm15166282

**Published:** 2026-08-13

**Authors:** Ahmet Demirel, Bora İnceöz, Ali Kaan Güren, Burak Paçacı, Erkam Kocaaslan, Mustafa Alperen Tunç, Fırat Akagündüz, Emek Kaya, Canan Çimşit, Nazım Can Demircan, İbrahim Vedat Bayoğlu

**Affiliations:** Division of Medical Oncology, Department of Internal Medicine, Marmara University Faculty of Medicine, Marmara University Pendik Training and Research Hospital, Istanbul 34854, Türkiye; boraimedicine@gmail.com (B.İ.); alikaanguren@gmail.com (A.K.G.); drpacaci@gmail.com (B.P.); erkamkocaaslan@gmail.com (E.K.); m.alperen.tunc@gmail.com (M.A.T.); fratakagunduz0@gmail.com (F.A.); emek.kaya.98@gmail.com (E.K.); canancimsit@gmail.com (C.Ç.); ncdemircan@gmail.com (N.C.D.); dr.vebay@gmail.com (İ.V.B.)

**Keywords:** pancreatic ductal adenocarcinoma, FOLFIRINOX, body composition, computed tomography, sarcopenia, skeletal muscle index, visceral adipose tissue, cachexia, prognosis

## Abstract

**Background:** Computed tomography (CT)-derived body composition has emerged as a promising prognostic biomarker in pancreatic ductal adenocarcinoma (PDAC). However, most previous studies have relied on baseline measurements and evaluated either resected or metastatic disease separately. We aimed to investigate the prognostic significance of both baseline and longitudinal CT-derived body composition changes in clinically distinct but therapeutically homogeneous cohorts of patients with PDAC receiving FOLFIRINOX. **Methods:** This retrospective single-center study included 98 consecutive patients with histologically confirmed PDAC treated with FOLFIRINOX between 2018 and 2025. Forty-seven patients underwent curative-intent resection followed by adjuvant modified FOLFIRINOX, whereas 51 patients with unresectable metastatic disease received first-line FOLFIRINOX. Skeletal muscle index (SMI) and visceral adipose tissue (VAT) were quantified on serial CT scans obtained at the third lumbar vertebral level before treatment and during therapy. Follow-up CT scans suitable for longitudinal body composition analysis were available for 46 of 51 patients (90.2%) in the metastatic cohort. Overall survival (OS), disease-free survival (DFS), and progression-free survival (PFS) were estimated using the Kaplan–Meier method. Univariable and multivariable Cox proportional hazards regression analyses were performed to identify independent prognostic factors. **Results:** Baseline CT-derived body composition parameters were not independently associated with survival in either cohort. In the resected cohort, preservation of visceral adiposity during treatment (follow-up VAT > 100 cm^2^) independently predicted improved OS in a multivariable model including three covariates (HR 0.460, 95% CI 0.220–0.960; *p* = 0.039). Median DFS and OS were 11.7 months (95% CI 6.5–16.9) and 20.9 months (95% CI 12.1–29.7), respectively. In the metastatic cohort, treatment-related skeletal muscle loss (ΔSMI) independently predicted inferior OS in a multivariable model including four covariates (HR 0.949, 95% CI 0.913–0.986; *p* = 0.008), while lung metastasis was also independently associated with worse survival (HR 5.792, 95% CI 1.880–17.841; *p* = 0.002). Median PFS and OS were 9.7 months (95% CI 8.0–11.4) and 12.7 months (95% CI 9.9–15.5), respectively. **Conclusions:** Longitudinal CT-derived body composition changes may provide additional prognostic information beyond baseline measurements in patients with PDAC receiving FOLFIRINOX. Preservation of visceral adiposity was associated with improved survival following curative-intent resection, whereas greater treatment-related skeletal muscle loss was associated with poorer survival in metastatic disease. These findings suggest that the prognostic relevance of body composition may vary according to disease stage and should be considered hypothesis-generating pending validation in larger prospective multicenter studies.

## 1. Introduction

Pancreatic ductal adenocarcinoma (PDAC) remains one of the most aggressive solid malignancies and is currently the seventh leading cause of cancer-related death worldwide. Despite continuous advances in surgical techniques, systemic chemotherapy, perioperative management, and supportive care, the overall 5-year survival rate remains below 15%, reflecting the aggressive biology of the disease, delayed diagnosis, early metastatic dissemination, and intrinsic resistance to therapy. Although FOLFIRINOX has substantially improved survival in both resected and metastatic PDAC, considerable heterogeneity in clinical outcomes persists even among patients receiving identical treatment regimens, suggesting that tumor burden alone cannot fully explain prognosis [1,2,3].

Beyond its oncological aggressiveness, PDAC is one of the malignancies most profoundly associated with cancer cachexia. Approximately 70–80% of patients experience clinically significant involuntary weight loss during the course of their disease, while nearly one-third ultimately die from complications related to cachexia rather than direct tumor progression. Cancer cachexia is a multifactorial metabolic syndrome characterized by progressive skeletal muscle depletion, with or without loss of adipose tissue, that cannot be completely reversed by conventional nutritional support. The syndrome is driven by complex interactions between tumor-derived factors, systemic inflammation, altered energy metabolism, reduced nutritional intake, endocrine dysregulation, and accelerated proteolysis. Consequently, alterations in skeletal muscle and adipose tissue are increasingly recognized as biological manifestations of tumor-host interactions rather than simple indicators of nutritional status [4,5,6,7,8].

Computed tomography (CT) has become the reference standard for body composition assessment because it enables objective and reproducible quantification of skeletal muscle area (SMA), skeletal muscle index (SMI), visceral adipose tissue (VAT), and subcutaneous adipose tissue (SAT) using routinely acquired abdominal imaging. Since serial CT examinations are already incorporated into routine response assessment during systemic therapy, longitudinal evaluation of body composition can be performed without additional radiation exposure, imaging procedures, or healthcare costs. Accordingly, CT-derived body composition analysis has emerged as a practical imaging biomarker capable of providing prognostic information beyond conventional clinicopathological variables [5,6,7,9].

Accumulating evidence suggests that sarcopenia and visceral adiposity influence postoperative outcomes, chemotherapy tolerance, treatment-related toxicity, and survival in patients with pancreatic cancer. However, published findings remain inconsistent. While several studies have reported baseline sarcopenia as an adverse prognostic factor, others have failed to demonstrate an independent association after adjustment for clinical and pathological variables. Likewise, the prognostic significance of visceral adiposity remains controversial, with studies reporting both favorable and unfavorable effects depending on patient selection and treatment setting. These discrepancies are likely attributable to differences in study design, CT measurement methodology, sarcopenia definitions, and heterogeneous treatment populations [7,10,11,12,13,14].

Importantly, four major knowledge gaps remain. First, most previous studies have focused exclusively on pretreatment body composition, whereas longitudinal changes during systemic therapy may better reflect the biological consequences of tumor progression and treatment-related metabolic adaptation. Second, relatively few investigations have simultaneously evaluated skeletal muscle and adipose tissue dynamics throughout treatment. Third, many published cohorts included patients receiving heterogeneous chemotherapy regimens, making it difficult to distinguish treatment-related effects from disease biology. Finally, resected and metastatic PDAC have generally been investigated independently, and whether the prognostic significance of dynamic body composition differs according to disease stage remains largely unknown [10,11,13,15,16,17].

FOLFIRINOX was selected because it is the current standard-of-care regimen for medically fit patients with both resected and metastatic PDAC. Restricting the study population to patients receiving the same systemic treatment minimized treatment-related heterogeneity and enabled a more accurate evaluation of stage-specific body composition changes.

FOLFIRINOX has become the standard-of-care chemotherapy regimen for medically fit patients with both resected and metastatic PDAC following the landmark PRODIGE 4 and PRODIGE 24 trials [2,3]. Evaluation of body composition within a homogeneous FOLFIRINOX-treated population minimizes treatment-related heterogeneity and provides a unique opportunity to investigate the biological relationship between longitudinal body composition changes and oncological outcomes. Furthermore, comparison of patients treated with the same systemic regimen in distinct clinical settings may provide novel insights into stage-specific host metabolic responses during pancreatic cancer progression [2,11,13,15].

Therefore, the present study was designed to address these four important knowledge gaps by: evaluating both baseline and longitudinal CT-derived body composition parameters; simultaneously assessing skeletal muscle and adipose tissue dynamics; restricting the analysis to a therapeutically homogeneous population receiving FOLFIRINOX; and directly comparing stage-specific prognostic patterns between resected and metastatic PDAC. We hypothesized that longitudinal body composition parameters would provide greater prognostic information than baseline measurements and that the dominant prognostic body composition marker would differ according to disease stage.

## 2. Materials and Methods

### 2.1. Study Design and Patient Population

This retrospective, single-center observational study included patients with histologically confirmed pancreatic ductal adenocarcinoma (PDAC) who received FOLFIRINOX-based chemotherapy at Marmara University Pendik Training and Research Hospital between January 2018 and December 2025. Patients were stratified according to treatment setting into two independent cohorts: (1) a resected cohort receiving adjuvant modified FOLFIRINOX following curative-intent surgery and (2) a metastatic cohort receiving first-line FOLFIRINOX for unresectable metastatic disease.

Eligible patients were required to be 18 years of age or older, have histologically confirmed PDAC, receive FOLFIRINOX as the initial treatment strategy within their respective cohort, undergo baseline contrast-enhanced abdominal computed tomography (CT) before treatment initiation, and have at least one follow-up CT examination available for longitudinal body composition analysis. Patients with a concurrent active malignancy, unavailable or poor-quality CT images precluding body composition analysis, incomplete clinical data, or inadequate follow-up were excluded. Because the primary objective of the study was to evaluate longitudinal changes in body composition, only patients with both baseline and at least one evaluable follow-up CT examination were eligible for inclusion in the longitudinal analyses.

Because of the retrospective nature of the study and limitations of the historical screening records, the complete patient flow from all patients with PDAC treated with FOLFIRINOX during the study period could not be reliably reconstructed. Specifically, the exact numbers of patients excluded before final cohort assembly because of unavailable baseline CT, inadequate CT image quality, incomplete clinical data, concurrent malignancy, or death or clinical deterioration before the first follow-up assessment were not systematically recorded. Therefore, a complete screening and exclusion flow diagram could not be generated. The final study population comprised 47 patients in the resected cohort and 51 patients in the metastatic cohort; follow-up CT suitable for longitudinal body composition assessment was available for 46 of 51 patients in the metastatic cohort.

The study was conducted in accordance with the ethical principles of the Declaration of Helsinki and was approved by the Marmara University Faculty of Medicine Clinical Research Ethics Committee (Approval No. 09.2026.26-0443). The requirement for written informed consent was waived because of the retrospective nature of the study.

#### Treatment

Patients in the resected cohort underwent curative-intent pancreatic resection followed by adjuvant modified FOLFIRINOX according to institutional practice. Patients in the metastatic cohort received first-line FOLFIRINOX as the standard systemic treatment for unresectable metastatic disease.

Dose reductions, treatment delays, and treatment discontinuation were implemented at the discretion of the treating medical oncologist based on hematologic and non-hematologic toxicities, Eastern Cooperative Oncology Group (ECOG) performance status, and the patient’s overall clinical condition.

Tumor response was assessed according to the Response Evaluation Criteria in Solid Tumors (RECIST) version 1.1 as part of routine institutional clinical practice.

### 2.2. CT Acquisition and Body Composition Analysis

Contrast-enhanced abdominal CT examinations were performed using a 256-detector CT scanner (Philips Brilliance ICT 256; Philips Medical Systems, Cleveland, OH, USA). Images were acquired using a tube voltage of 120 kVp, whereas tube current (mAs) was automatically adjusted according to patient characteristics and institutional imaging protocols. Images were reconstructed with a slice thickness of 5 mm and an overlap interval of 5 mm.

For the resected cohort, baseline CT examinations referred to postoperative CT scans obtained within four weeks before initiation of adjuvant FOLFIRINOX therapy. For the metastatic cohort, baseline CT examinations referred to pretreatment CT scans obtained within four weeks before initiation of first-line FOLFIRINOX therapy. Follow-up CT examinations used for longitudinal body composition analysis were routinely performed approximately 3–4 months after treatment initiation, corresponding to the first standardized radiological response assessment in routine clinical practice. The median interval between baseline and follow-up CT examinations was 101 days (IQR, 83–131 days) in the resected cohort and 98.5 days (IQR, 85–114 days) in the metastatic cohort.

Body composition analysis was performed using the three-dimensional image analysis module integrated into Synapse PACS (Version 7.4.010; FUJIFILM Healthcare Americas Corporation, Lexington, MA, USA). All CT measurements were performed on axial images acquired in the supine position using a standardized institutional protocol.

Skeletal muscle area (SMA), visceral adipose tissue (VAT), and subcutaneous adipose tissue (SAT) were quantified on a single axial CT image obtained at the level of the third lumbar vertebra (L3) [6]. Skeletal muscle measurements included the psoas, erector spinae, quadratus lumborum, transversus abdominis, internal and external oblique, and rectus abdominis muscles.

Skeletal muscle area (SMA), visceral adipose tissue area (VAT), and subcutaneous adipose tissue area (SAT) were measured in cm^2^. Skeletal muscle index (SMI), visceral adipose tissue index (VATI), and subcutaneous adipose tissue index (SATI) were calculated by normalizing the corresponding tissue area to the square of patient height (cm^2^/m^2^).

Baseline and follow-up CT examinations for each patient were analyzed using the same software platform and identical measurement protocol. To ensure measurement reliability, all body composition measurements were initially performed by an experienced radiologist and subsequently reviewed by a second experienced radiologist. Both radiologists were blinded to patients’ clinical characteristics, treatment response, survival outcomes, and the study hypothesis throughout image analysis. Any discrepancies identified during the review process were resolved by consensus, and the final agreed measurements were used for statistical analyses.

Longitudinal body composition changes were expressed as percentage changes between baseline and follow-up measurements using the following equations:

SMI = \frac(SMA\ (cm^2^)(height^2^\ (m^2^))

VATI = \frac{VAT\ (cm^2^))(height^2^\ (m^2^))

SATI = \frac(SAT\ (cm^2^))(height^2^\ (m^2^))

\Delta Parameter (%) = \frac(Follow\text(-)up-Baseline)(Baseline) \times100

Accordingly, ΔSMI (%), ΔVATI (%), and ΔSATI (%) represented treatment-related percentage changes in skeletal muscle, visceral adipose tissue, and subcutaneous adipose tissue, respectively.

### 2.3. Definitions

High visceral adiposity was defined as a visceral adipose tissue (VAT) area > 100 cm^2^, a threshold previously used in CT-based oncologic body composition studies to characterize visceral obesity. Because no universally established PDAC-specific VAT cutoff is available, this threshold was applied as a pragmatic, literature-based definition [18,19]. Low skeletal muscle index (SMI) (sarcopenia) was defined as an SMI < 50 cm^2^/m^2^ for men and <39 cm^2^/m^2^ for women according to the sex-specific cut-off values proposed by Martin et al. These thresholds are among the most widely validated CT-based definitions of sarcopenia in patients with gastrointestinal malignancies, including pancreatic ductal adenocarcinoma, and have been extensively adopted in oncologic body composition research [5,7].

#### Study Outcomes

The primary endpoint of the study was overall survival (OS). Secondary endpoints included progression-free survival (PFS) in the metastatic cohort, treatment completion, dose reduction during FOLFIRINOX therapy, and the prognostic significance of baseline and longitudinal CT-derived body composition parameters.

For the resected cohort, overall survival (OS) was defined as the time interval between the date of diagnosis and death from any cause or the date of the last follow-up. Disease-free survival (DFS) was defined as the time interval between the date of curative-intent surgery and the first documented disease recurrence, death from any cause, or the date of the last follow-up, whichever occurred first.

For the metastatic cohort, overall survival (OS) was defined as the time interval between initiation of FOLFIRINOX therapy and death from any cause or the date of the last follow-up. Progression-free survival (PFS) was defined as the time interval between initiation of systemic therapy and radiologically confirmed disease progression or death from any cause.

### 2.4. Statistical Analysis

Continuous variables are presented as mean ± standard deviation (SD) or median with interquartile range (IQR), as appropriate. Categorical variables are summarized as frequencies and percentages.

Comparisons between categorical variables were performed using the Pearson χ^2^ test or Fisher’s exact test, as appropriate. Continuous variables were compared using the independent-samples *t*-test or Mann–Whitney U test, according to the distribution and characteristics of the data.

Overall survival (OS) and progression-free survival (PFS) were estimated using the Kaplan–Meier method and compared using the log-rank test. Variables associated with survival were initially evaluated using univariable Cox proportional hazards regression analysis. Variables demonstrating a univariable association with survival (*p* < 0.10), together with clinically relevant covariates selected a priori, were subsequently entered into multivariable Cox proportional hazards regression models to identify independent prognostic factors. Accordingly, baseline CA19-9, which showed a borderline association with survival in univariable analysis (*p* = 0.074), was included in the multivariable model because it met the prespecified *p* < 0.10 inclusion criterion. Hazard ratios (HRs) and corresponding 95% confidence intervals (CIs) were reported. To address potential survivorship (immortal time) bias related to the requirement for follow-up CT imaging, a landmark sensitivity analysis was additionally performed. In this analysis, the date of the follow-up CT examination was defined as the landmark (time zero), and OS was recalculated from the follow-up CT date to death or last follow-up. Multivariable Cox proportional hazards regression models were then repeated using the same covariates as in the primary models.

No formal sample size calculation or a priori power analysis was performed because this was a retrospective observational study. All consecutive eligible patients treated during the predefined study period who met the inclusion criteria were included in the analysis.

Missing data were handled using a complete-case approach. Patients without evaluable follow-up CT examinations or with CT images of insufficient quality for body composition analysis were excluded from longitudinal analyses. No imputation of missing data was performed. The number of evaluable patients was reported separately for each longitudinal body composition parameter and survival model.

Given the exploratory nature of this study and the relatively limited sample size, no formal adjustment for multiple comparisons (e.g., Bonferroni correction) was performed. The results were interpreted cautiously with emphasis on effect sizes, confidence intervals, and consistency across analyses.

All statistical analyses were performed using IBM SPSS Statistics version 26.0 (IBM Corp., Armonk, NY, USA). All statistical tests were two-sided, and a *p*-value < 0.05 was considered statistically significant.

The study was designed, conducted, and reported in accordance with the Strengthening the Reporting of Observational Studies in Epidemiology (STROBE) statement. The completed STROBE checklist is provided in the Supplementary Materials.

## 3. Results

### 3.1. Patient Characteristics

A total of 98 patients with histologically confirmed pancreatic ductal adenocarcinoma (PDAC) treated with FOLFIRINOX were included in the study. Of these, 47 patients underwent curative-intent resection followed by adjuvant modified FOLFIRINOX (resected cohort), whereas 51 patients with unresectable metastatic disease received first-line FOLFIRINOX (metastatic cohort).

Baseline demographic and clinical characteristics are summarized in Table 1. Patients in the metastatic cohort were older than those in the resected cohort (median age, 64.0 vs. 57.0 years) and were more frequently male (72.5% vs. 51.1%). Body mass index was comparable between the two cohorts. As expected, performance status differed considerably according to disease stage, with 91.5% of patients in the resected cohort presenting with an ECOG performance status of 0 compared with 49.0% in the metastatic cohort.

Despite these clinical differences, baseline body composition was remarkably similar between the two groups. High visceral adiposity (VAT > 100 cm^2^) was observed in 66.0% of resected patients and 58.8% of metastatic patients, indicating comparable visceral fat distribution before treatment initiation.

### 3.2. Body Composition Characteristics

Baseline and longitudinal body composition characteristics are presented in Table 2.

At baseline, median skeletal muscle index (SMI) was comparable between the resected and metastatic cohorts (46.4 vs. 45.4 cm^2^/m^2^). However, the prevalence of sarcopenia was substantially higher in the metastatic cohort (64.7% vs. 46.8%). Likewise, high visceral adiposity (VAT > 100 cm^2^) was common in both treatment groups.

Serial CT assessment demonstrated progressive deterioration of body composition during FOLFIRINOX therapy in both cohorts. The proportion of patients with sarcopenia increased from 46.8% to 63.8% in the resected cohort and from 64.7% to 80.4% among evaluable metastatic patients. Similarly, the prevalence of VAT > 100 cm^2^ decreased from 66.0% to 31.9% in the resected cohort and from 58.8% to 41.3% in the metastatic cohort.

Analysis of longitudinal body composition changes demonstrated marked reductions in both skeletal muscle and adipose tissue during treatment. Median percentage skeletal muscle loss was comparable between cohorts (ΔSMI: −8.4% vs. −8.8%). In contrast, visceral adipose tissue depletion appeared more pronounced in resected patients, with a median ΔVATI of −43.2% compared with −26.3% in metastatic patients. Median ΔSATI was −43.0% and −39.5% in the resected and metastatic cohorts, respectively.

#### Treatment Characteristics and Survival Outcomes

Treatment characteristics and survival outcomes are summarized in Table 3. Among patients in the resected cohort, the median number of adjuvant FOLFIRINOX cycles was 10 (IQR, 6–12), and treatment completion was achieved in 18 patients (38.3%). Dose reductions were required in 32 patients (68.1%), including one dose reduction in 25 (53.2%) and two dose reductions in 7 (14.9%). Among the 28 patients who discontinued treatment prematurely, the most common reasons were disease progression (25.0%), poor performance status (17.9%), gastrointestinal toxicity (14.3%), patient preference (14.3%), neutropenia (7.1%), thrombocytopenia (7.1%), neuropathy (7.1%), and infection (7.1%). In the metastatic cohort, dose reductions occurred in 32 patients (62.7%), and 14 patients (27.5%) remained on first-line FOLFIRINOX treatment at the time of data cut-off.

Among patients in the resected cohort, the median disease-free survival (DFS) was 11.7 months (95% CI, 6.5–16.9) and the median overall survival (OS) was 20.9 months (95% CI, 12.1–29.7). In the metastatic cohort, the median progression-free survival (PFS) was 9.7 months (95% CI, 8.0–11.4) and the median overall survival (OS) was 12.7 months (95% CI, 9.9–15.5).

### 3.3. Prognostic Significance of CT-Derived Body Composition

The results of the univariable and multivariable Cox proportional hazards regression analyses for overall survival are summarized in Table 4, Table 5 and Table 6.

#### 3.3.1. Resected Cohort

Baseline CT-derived body composition parameters, including baseline skeletal muscle index (SMI), low baseline SMI, and baseline visceral adiposity (VAT > 100 cm^2^), were not significantly associated with overall survival in the univariable Cox regression analysis (Table 4).

**Figure 1:**
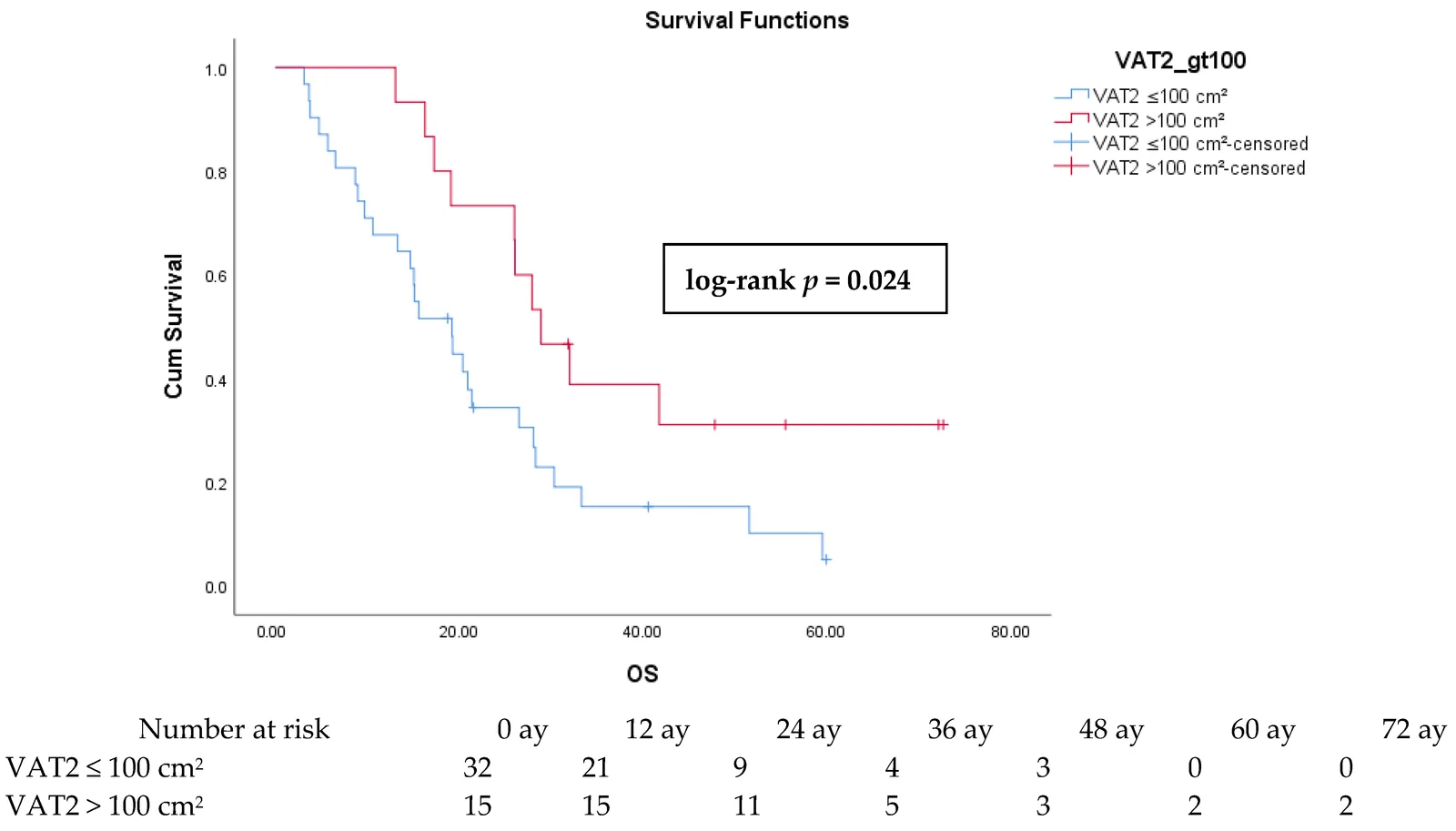
Patients with VAT2 > 100 cm^2^ had significantly longer overall survival compared with patients with VAT2 ≤ 100 cm^2^ (log-rank *p* = 0.024).

Among the longitudinal body composition parameters, preservation of visceral adiposity during treatment (follow-up VAT > 100 cm^2^) was significantly associated with improved overall survival (HR, 0.440; 95% CI, 0.212–0.916; *p* = 0.028). Kaplan–Meier analysis confirmed this finding, demonstrating significantly prolonged overall survival in patients with preserved visceral adiposity (log-rank *p* = 0.024; Figure 1). Although follow-up skeletal muscle area and ΔVATI showed trends toward improved survival, neither reached statistical significance.

In the multivariable Cox regression model adjusted for sex and adjuvant treatment completion, follow-up VAT > 100 cm^2^ remained an independent predictor of improved overall survival (HR, 0.460; 95% CI, 0.220–0.960; *p* = 0.039) (Table 6).

#### 3.3.2. Metastatic Cohort

The distribution of metastatic disease is summarized in Table 5. Liver metastases were present in 42 patients (82.4%), lymph node metastases in 33 (64.7%), peritoneal metastases in 8 (15.7%), lung metastases in 7 (13.7%), and a bone metastasis in 1 (2.0%). Data regarding the number of metastatic sites were available for 34 patients; among these, 6 (17.6%) had one metastatic site, 7 (20.6%) had two sites, and 21 (61.8%) had three or more metastatic sites.

Baseline CT-derived body composition parameters were not significantly associated with overall survival in the univariable Cox regression analysis (Table 5).

In contrast, longitudinal skeletal muscle loss was significantly associated with adverse survival outcomes. Each percentage decrease in skeletal muscle index during treatment (ΔSMI) was associated with poorer overall survival (HR, 0.961; 95% CI, 0.929–0.994; *p* = 0.022).

Among the clinicopathological variables, the presence of lung metastasis (HR, 3.174; 95% CI, 1.347–7.481; *p* = 0.008) was significantly associated with inferior overall survival, whereas baseline CA19-9 demonstrated a borderline association (*p* = 0.074) (Table 5).

In the multivariable Cox proportional hazards regression model adjusted for sex, lung metastasis, and baseline CA19-9, ΔSMI remained an independent predictor of overall survival (HR, 0.949; 95% CI, 0.913–0.986; *p* = 0.008). The presence of lung metastasis also remained independently associated with inferior overall survival (HR, 5.792; 95% CI, 1.880–17.841; *p* = 0.002) (Table 7).

To address potential survivorship (immortal time) bias related to the requirement for follow-up CT imaging, a landmark sensitivity analysis was performed using the follow-up CT date as time zero. In the resected cohort (n = 47; 38 deaths), follow-up VAT > 100 cm^2^ remained independently associated with improved OS (HR, 0.425; 95% CI, 0.203–0.887; *p* = 0.023). In the metastatic cohort (n = 41; 35 deaths), ΔSMI remained independently associated with OS (HR, 0.950; 95% CI, 0.914–0.988; *p* = 0.010). These findings were consistent with the direction and magnitude of the primary multivariable analyses.

### 3.4. Summary of the Main Findings

**Figure 2:**
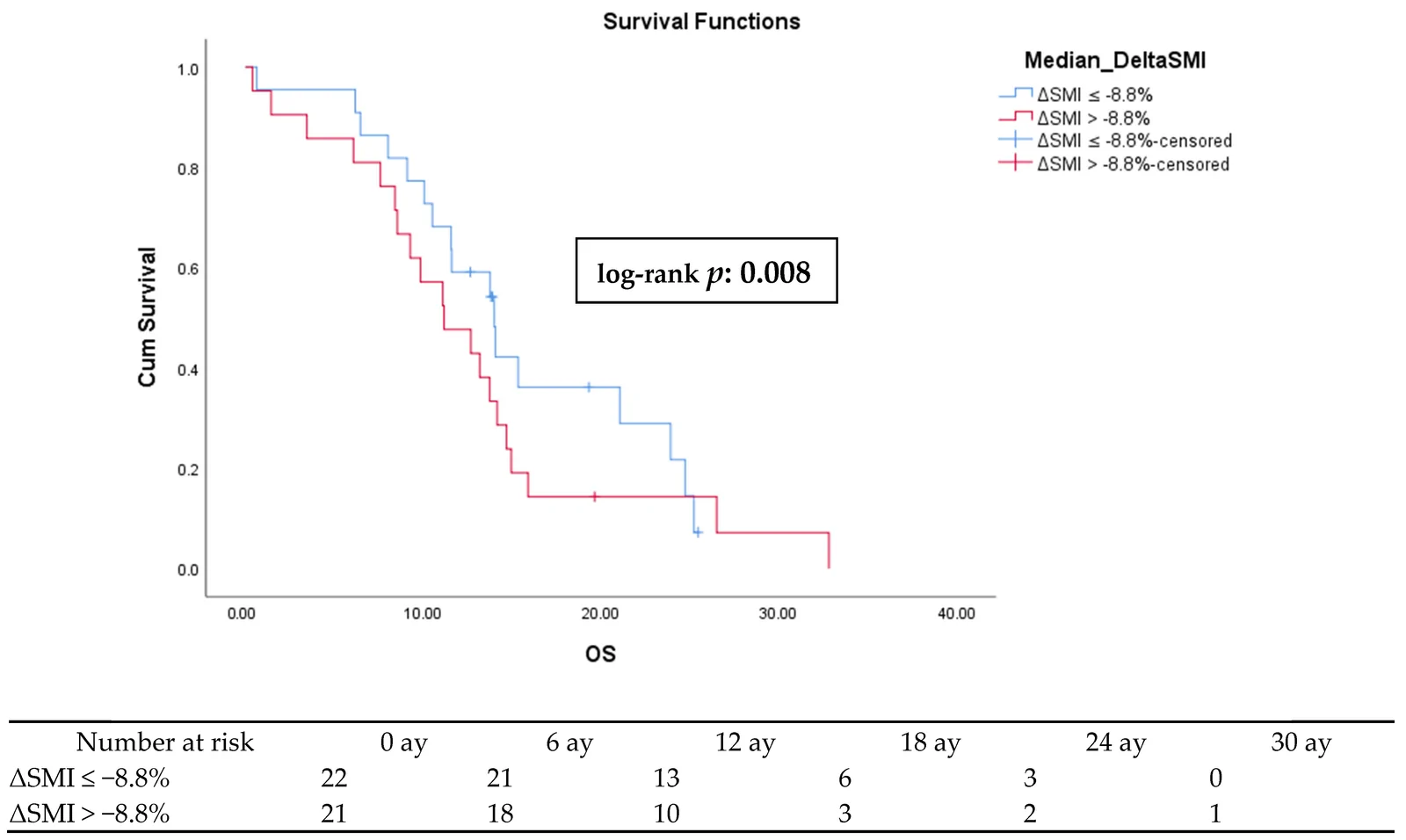
Kaplan–Meier overall survival according to treatment-related skeletal muscle loss (ΔSMI) in patients with metastatic pancreatic ductal adenocarcinoma receiving first-line FOLFIRINOX.

**Figure 3:**
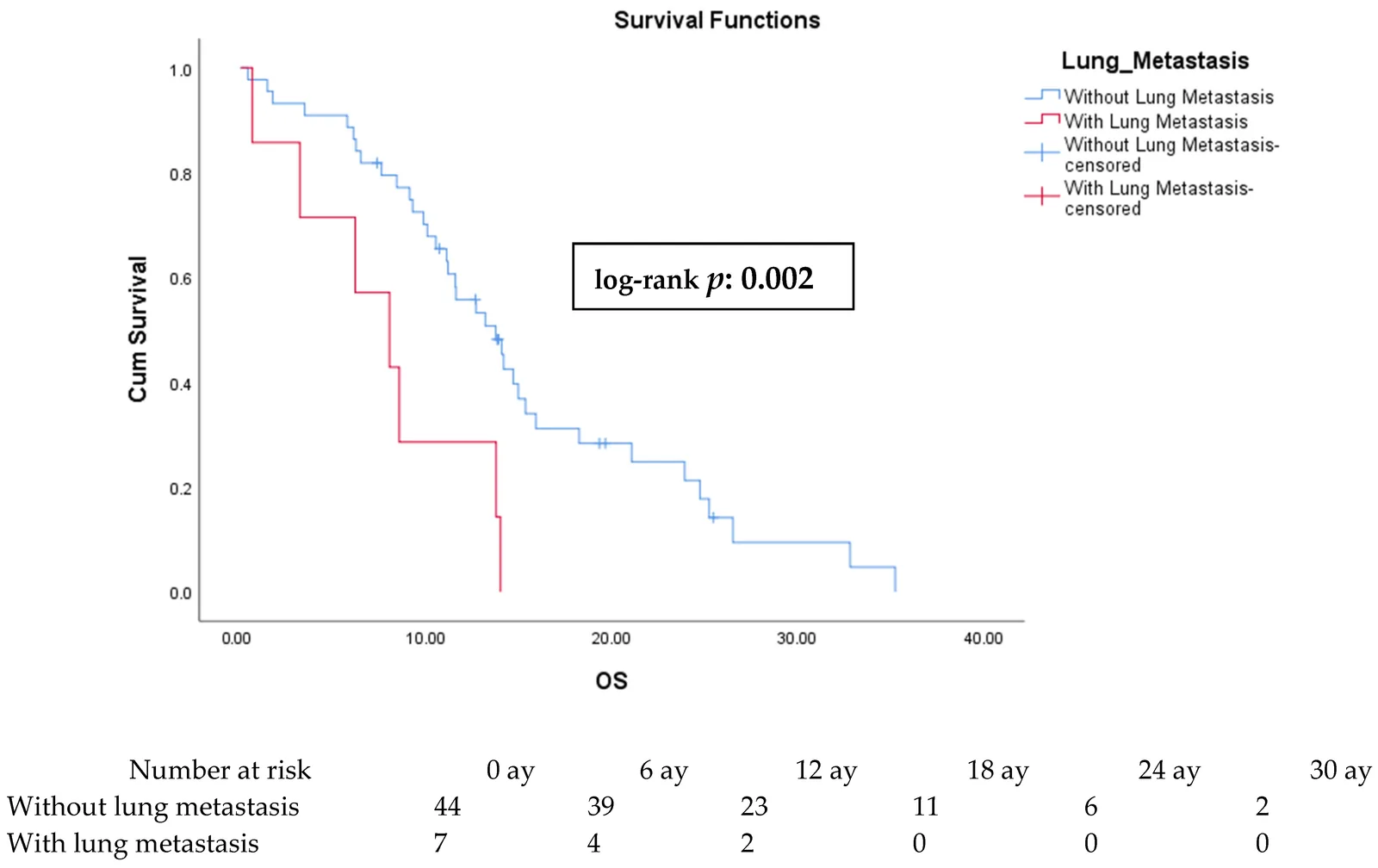
Kaplan–Meier overall survival curves stratified by lung metastasis status in the metastatic cohort. The presence of lung metastasis was associated with significantly inferior overall survival. Tick marks indicate censored patients.

Overall, baseline CT-derived body composition parameters were not independently associated with overall survival in either the resected or metastatic cohort. In contrast, longitudinal assessment of body composition identified distinct stage-specific prognostic markers. Preservation of visceral adiposity during adjuvant FOLFIRINOX independently predicted improved overall survival following curative-intent resection, whereas treatment-related skeletal muscle loss independently predicted poorer overall survival in metastatic disease(Figure 2). In addition, the presence of lung metastasis was associated with significantly inferior overall survival in the metastatic cohort (Figure 3). These findings suggest that longitudinal CT-derived body composition assessment provides greater prognostic value than baseline measurements and that the prognostic relevance of individual body composition parameters differs according to disease stage.

## 4. Discussion

The present study demonstrates that the prognostic significance of CT-derived body composition is not universal but rather depends on disease stage in patients with pancreatic ductal adenocarcinoma receiving FOLFIRINOX. Although baseline body composition parameters showed limited prognostic value in both treatment settings, longitudinal treatment-related alterations identified distinct stage-specific prognostic markers. Preservation of visceral adiposity independently predicted improved overall survival following curative-intent resection, whereas treatment-related skeletal muscle loss emerged as the strongest body composition predictor of survival in metastatic disease. These findings suggest that dynamic changes in host body composition provide substantially greater prognostic information than static pretreatment measurements and highlight the biological heterogeneity of cachexia across different stages of pancreatic cancer [4].

Interest in CT-derived body composition as a prognostic biomarker has increased considerably over the past decade. Numerous studies have demonstrated associations between sarcopenia, adipose tissue depletion, and inferior survival across multiple malignancies, particularly pancreatic cancer [7,20,21]. Nevertheless, the majority of previous investigations relied exclusively on baseline CT measurements obtained before treatment initiation [22]. While pretreatment sarcopenia undoubtedly reflects impaired physiological reserve, it represents only a single time point within an evolving metabolic process. In contrast, longitudinal body composition changes capture the dynamic interaction between tumor biology, systemic inflammation, nutritional status, treatment-related toxicity, and host adaptation throughout therapy. Consequently, serial CT assessment may better reflect the biological trajectory of disease than baseline measurements alone [4,8].

Our findings further support this concept. None of the baseline skeletal muscle or visceral adiposity parameters independently predicted survival in either cohort, whereas longitudinal alterations during treatment demonstrated clear prognostic significance. This observation suggests that baseline body composition represents constitutional host characteristics, whereas treatment-related changes more accurately reflect the cumulative biological effects of tumor progression and systemic therapy [4,8]. These findings are consistent with the growing concept that cancer cachexia is a dynamic and progressive syndrome rather than a static condition measurable at a single time point. Accordingly, repeated assessment of body composition during treatment may provide substantially greater prognostic information than pretreatment evaluation alone [8,23].

An additional strength of the present study is the use of two clinically distinct yet therapeutically homogeneous cohorts. Most previous studies evaluated either resected or metastatic pancreatic cancer separately, frequently including patients treated with heterogeneous chemotherapy regimens [21,22]. By restricting both cohorts to patients receiving FOLFIRINOX, treatment-related heterogeneity was minimized, allowing a more direct evaluation of the relationship between longitudinal body composition changes and survival [2,3]. More importantly, direct comparison between adjuvant and metastatic settings demonstrated that the prognostic relevance of individual body composition parameters differs according to disease stage, suggesting that host metabolic adaptation evolves throughout pancreatic cancer progression rather than remaining biologically constant [4].

Among patients who underwent curative-intent resection, preservation of visceral adiposity during adjuvant FOLFIRINOX emerged as the only independent body composition parameter associated with prolonged overall survival. Interestingly, baseline visceral adiposity showed no prognostic significance, whereas follow-up VAT remained independently associated with survival after adjustment for sex and treatment completion. Preserved follow-up VAT may reflect a patient’s metabolic resilience during systemic therapy; however, this interpretation remains hypothesis-generating given the observational nature of the present study [7,8,24].

Traditionally, visceral adiposity has been regarded as metabolically unfavorable because of its association with insulin resistance, chronic inflammation, and cardiometabolic disease [25]. However, in patients with advanced malignancies, the biological significance of visceral fat appears considerably more complex. Adipose tissue represents the body’s largest energy reservoir and plays a central role in maintaining metabolic homeostasis during periods of increased catabolic stress. During cancer treatment, progressive depletion of visceral fat may therefore reflect sustained negative energy balance, systemic inflammation, and activation of cachexia-related pathways rather than simply reduced adiposity. Preservation of visceral adipose tissue may indicate maintenance of metabolic reserve sufficient to withstand the physiological stress imposed by both major pancreatic surgery and intensive multi-agent chemotherapy [4,8,24].

Several previous studies have investigated the relationship between visceral adiposity and outcomes in pancreatic cancer, although their conclusions have remained inconsistent [21,22,26]. Whereas some authors reported obesity or increased visceral fat to be associated with higher postoperative morbidity and inferior oncological outcomes, others demonstrated neutral or even favorable survival associations after adjustment for body composition and nutritional status. These discrepancies likely reflect differences in patient populations, disease stage, treatment strategy, and timing of CT assessment [4,8,22,27,28]. Importantly, most published studies evaluated only pretreatment adiposity. In contrast, our findings indicate that preservation of visceral adiposity during treatment provides substantially greater prognostic information than baseline visceral fat alone, emphasizing that dynamic metabolic adaptation may be biologically more relevant than constitutional body composition [4,8].

In contrast to the resected cohort, skeletal muscle dynamics rather than adipose tissue determined prognosis in metastatic disease. Treatment-related decline in skeletal muscle index independently predicted inferior overall survival even after adjustment for metastatic burden and serum CA19-9 levels. This observation highlights the dominant role of progressive muscle wasting in advanced pancreatic cancer and is biologically consistent with the concept of cancer cachexia as a systemic catabolic syndrome [4,8]. Skeletal muscle is increasingly recognized not merely as a mechanical tissue but also as an endocrine organ involved in immune regulation, glucose metabolism, mitochondrial function, and whole-body protein homeostasis. Progressive skeletal muscle depletion therefore reflects profound metabolic failure that extends well beyond simple loss of lean body mass [24,29].

The biological mechanisms underlying treatment-related muscle loss are multifactorial [4,8]. Systemic inflammation and cancer-related metabolic alterations may contribute to progressive skeletal muscle depletion in patients with advanced pancreatic cancer [4,8,24,30]. Reduced nutritional intake, pancreatic exocrine insufficiency, insulin resistance, decreased physical activity, and chemotherapy-related toxicities may further accelerate skeletal muscle loss. Consequently, longitudinal muscle loss during treatment may reflect the combined effects of tumor progression, host catabolism, and treatment-related metabolic stress [31].

Our findings therefore support the emerging concept that body composition biomarkers should not be interpreted uniformly across all stages of pancreatic cancer. Instead, different body compartments may predominate according to the underlying biological context [8,24]. Following curative-intent surgery, preservation of visceral adipose tissue appears to reflect adequate metabolic reserve during recovery and adjuvant chemotherapy. Conversely, in metastatic disease, where systemic inflammation and cachexia become dominant drivers of outcome, progressive skeletal muscle wasting appears to be the principal body composition determinant of survival [8]. This stage-specific divergence may explain the inconsistent findings reported in previous body composition studies that combined heterogeneous patient populations or analyzed only a single disease stage [21,22].

From a clinical perspective, our findings have several important implications. Routine CT examinations are already performed during FOLFIRINOX treatment for response assessment, making longitudinal body composition analysis feasible without additional imaging, radiation exposure, or healthcare costs [5,7]. Integration of automated CT-derived body composition assessment into routine oncological practice could therefore provide valuable prognostic information beyond conventional clinical variables. Identification of patients experiencing rapid skeletal muscle depletion or marked visceral fat loss may facilitate earlier implementation of individualized nutritional support, structured exercise programs, pancreatic enzyme replacement when indicated, and multidisciplinary supportive care interventions [8,32]. Rather than serving solely as prognostic biomarkers, longitudinal body composition measurements may represent actionable indicators of host vulnerability during systemic treatment [33].

The present study possesses several methodological strengths. First, both cohorts received a homogeneous FOLFIRINOX regimen, minimizing treatment-related heterogeneity that has limited interpretation in many previous body composition studies [21,22]. Second, we evaluated both baseline and longitudinal CT-derived body composition parameters using standardized imaging protocols with blinded radiological assessment, allowing a comprehensive evaluation of dynamic host metabolic changes during treatment. Third, inclusion of both resected and metastatic cohorts enabled direct comparison of stage-specific prognostic patterns within the same disease entity, an approach that has rarely been explored in previous investigations. Finally, all body composition measurements were performed using routinely acquired clinical CT examinations, enhancing the potential applicability of our findings to everyday oncological practice [5].

Several limitations should also be acknowledged. The retrospective single-center design introduces the possibility of selection bias and limits the generalizability of our findings. In addition, the total number of patients initially screened, and the exact numbers excluded for individual eligibility criteria could not be reliably reconstructed from the available retrospective records, precluding a detailed assessment of the patient-selection process. Consequently, the number and characteristics of patients excluded because of unavailable or inadequate baseline imaging, incomplete clinical data, concurrent malignancy, or early death or clinical deterioration before follow-up assessment could not be quantified. This limits our ability to determine the magnitude and direction of potential selection bias and may reduce the generalizability of the study population. The relatively modest sample size, particularly for subgroup analyses, may have reduced statistical power for detecting weaker associations and contributed to wide confidence intervals for certain clinicopathological variables. Furthermore, the requirement for at least one evaluable follow-up CT examination may have introduced survivorship (immortal time) bias by excluding patients who experienced rapid disease progression, early death, or clinical deterioration before the first scheduled restaging scan. Consequently, the longitudinal analyses were necessarily restricted to patients who survived long enough and remained clinically stable enough to undergo repeat imaging, which should be considered when interpreting our findings. To mitigate this concern, we performed a landmark sensitivity analysis using the follow-up CT date as time zero. The principal longitudinal associations remained significant in both cohorts, supporting the robustness of the primary findings. Nevertheless, residual survivorship bias cannot be completely excluded because patients who died or clinically deteriorated before follow-up imaging were not represented in the longitudinal analyses. Accordingly, despite the consistency observed in the landmark sensitivity analysis, potential survivorship (immortal time) bias remains an important limitation of the present study and should be considered when interpreting the magnitude of the observed survival associations. Therefore, these findings should be regarded as exploratory and hypothesis-generating rather than as establishing definitive or causal prognostic effects. In addition, body composition was assessed at two predefined time points rather than through serial longitudinal measurements throughout the entire treatment course. Furthermore, although several established prognostic factors, including pathological characteristics in the resected cohort (e.g., nodal status, resection margin, lymphovascular and perineural invasion) and disease-burden variables in the metastatic cohort (e.g., metastatic distribution, inflammatory status, treatment response, and subsequent therapies), are known to influence survival, not all of these variables could be incorporated simultaneously into the multivariable models because of the relatively limited sample size and the need to avoid model overfitting. Therefore, residual confounding cannot be completely excluded, and our findings should be interpreted as hypothesis-generating. In addition, detailed treatment-related variables, including relative dose intensity, treatment delays, and standardized CTCAE toxicity grades, were not consistently available because of the retrospective design. Consequently, the relationship between longitudinal body composition changes and chemotherapy tolerance could not be comprehensively evaluated. We also lacked detailed information regarding nutritional interventions, pancreatic exocrine insufficiency, systemic inflammatory cytokines, body weight trajectories, physical activity, and muscle function, all of which may influence body composition dynamics independently of tumor progression [8,32]. These factors should be addressed in future prospective investigations.

Despite these limitations, our findings contribute to the growing evidence that longitudinal body composition assessment provides clinically meaningful prognostic information beyond conventional baseline measurements [8,23]. Importantly, our results suggest that the prognostic significance of individual body composition compartments is not uniform across pancreatic cancer but instead varies according to disease stage. This stage-specific pattern may partially explain the conflicting results reported by previous studies that combined heterogeneous patient populations or relied exclusively on pretreatment CT measurements [8,21,22].

Future prospective multicenter studies incorporating serial CT-based body composition analysis, nutritional assessment, inflammatory biomarkers, and functional performance measures are warranted to validate these findings [8,32]. Integration of artificial intelligence-assisted automated body composition analysis into routine radiological workflows may further facilitate widespread clinical implementation and enable real-time risk stratification during systemic therapy [5,34]. Moreover, interventional studies are needed to determine whether preservation of skeletal muscle and visceral adipose tissue through individualized nutritional, pharmacological, or exercise-based strategies can improve survival and treatment tolerance in patients receiving FOLFIRINOX [4,32].

Overall, the present study suggests that longitudinal assessment of treatment-related body composition changes may provide additional prognostic information beyond static baseline measurements. Rather than representing a universal prognostic biomarker, CT-derived body composition may have stage-specific prognostic relevance in pancreatic ductal adenocarcinoma. Preservation of visceral adiposity was associated with favorable survival following curative-intent resection, whereas greater treatment-related skeletal muscle loss was associated with poorer survival in metastatic disease. These findings highlight the potential importance of considering disease stage when interpreting body composition parameters and support further prospective investigation of longitudinal CT-derived body composition assessment in patients with pancreatic ductal adenocarcinoma [8,32]. Nevertheless, potential survivorship (immortal time) bias related to the requirement for evaluable follow-up imaging cannot be completely excluded, despite the consistency of the landmark sensitivity analysis. Therefore, these findings should be considered exploratory and hypothesis-generating and require validation in larger prospective studies.

## Figures and Tables

**Table 1:** Baseline Characteristics of the Resected and Metastatic Pancreatic Adenocarcinoma Cohorts.

Variable	Resected (n = 47)	Metastatic (n = 51)
Age, years, mean ± SD	58.5 ± 8.4	62.5 ± 8.7
Age, median (IQR)	57.0 (54.5–65.0)	64.0 (57.0–69.0)
Male sex, n (%)	24 (51.1)	37 (72.5)
Female sex, n (%)	23 (48.9)	14 (27.5)
BMI, kg/m^2^, mean ± SD	25.3 ± 3.7	24.0 ± 3.7
BMI, median (IQR)	25.4 (22.7–27.2)	23.8 (21.0–26.0)
ECOG 0, n (%)	43 (91.5)	25 (49.0)
ECOG 1, n (%)	4 (8.5)	22 (43.1)
ECOG 2, n (%)	—	4 (7.8)
Baseline VAT > 100 cm^2^, n (%)	31 (66.0)	30 (58.8)
Baseline VAT ≤ 100 cm^2^, n (%)	16 (34.0)	21 (41.2)

**Table 2:** Body Composition Characteristics Before and During Treatment.

Variable	Resected (n = 47)	Metastatic (n = 51)
Baseline body composition		
Skeletal muscle area (SMA), cm^2^, median (IQR)	123.9 (101.3–147.4)	130.5 (114.0–150.9)
Skeletal muscle index (SMI), cm^2^/m^2^, median (IQR)	46.4 (40.4–51.1)	45.4 (41.0–50.5)
Low SMI (sarcopenia), n (%)	22 (46.8)	33 (64.7)
Normal SMI, n (%)	25 (53.2)	18 (35.3)
VAT > 100 cm^2^, n (%)	31 (66.0)	30 (58.8)
VAT ≤ 100 cm^2^, n (%)	16 (34.0)	21 (41.2)
Follow-up body composition		
Skeletal muscle area (SMA), cm^2^, median (IQR)	111.6 (95.9–133.1)	115.2 (105.1–129.2)
Skeletal muscle index (SMI), cm^2^/m^2^, median (IQR)	41.7 (35.4–46.0)	41.1 (37.0–44.8)
Low SMI (sarcopenia), n (%)	30 (63.8)	37/46 (80.4)
Normal SMI, n (%)	17 (36.2)	9/46 (19.6)
VAT > 100 cm^2^, n (%)	15 (31.9)	19/46 (41.3)
VAT ≤ 100 cm^2^, n (%)	32 (68.1)	27/46 (58.7)
Treatment-related changes		
ΔSMI (%), median (IQR)	−8.4 (−14.4 to −3.0)	−8.8 (−14.8 to −3.0) †
ΔVATI (%), median (IQR)	−43.2 (−62.7 to −7.5)	−26.3 (−44.3 to −8.4) ‡
ΔSATI (%), median (IQR)	−43.0 (−58.5 to −13.4)	−39.5 (−58.3 to −12.2) ‡

Abbreviations: SMA, skeletal muscle area; SMI, skeletal muscle index; VAT, visceral adipose tissue; VATI, visceral adipose tissue index; SATI, subcutaneous adipose tissue index; IQR, interquartile range. Footnotes: Low SMI was defined as SMI < 50 cm^2^/m^2^ for men and <39 cm^2^/m^2^ for women. Follow-up CT was available for 46 metastatic patients. † ΔSMI was available for 43 metastatic patients. ‡ ΔVATI and ΔSATI were available for 46 metastatic patients.

**Table 3:** Treatment Exposure and Survival Outcomes.

Variable	Resected (n = 47)	Metastatic (n = 51)
Treatment characteristics		
Dose reduction, n (%)	32 (68.1)	32 (62.7)
No dose reduction, n (%)	15 (31.9)	—
One dose reduction, n (%)	25 (53.2)	—
Two dose reductions, n (%)	7 (14.9)	—
Adjuvant treatment completion, n (%)	18 (38.3)	—
Median number of FOLFIRINOX cycles, median (IQR)	10 (6–12)	—
Ongoing first-line treatment at data cut-off, n (%)	—	14 (27.5)
Reasons for treatment discontinuation †		
Disease progression, n	7	—
Poor performance status, n	5	—
Gastrointestinal toxicity, n	4	—
Patient preference, n	4	—
Neutropenia, n	2	—
Neuropathy, n	2	—
Infection, n	2	—
Thrombocytopenia, n	2	—
Survival outcomes		
Median DFS, months (95% CI)	11.7 (6.5–16.9)	—
Median PFS, months (95% CI)	—	9.7 (8.0–11.4)
Median OS, months (95% CI)	20.9 (12.1–29.7)	12.7 (9.9–15.5)

† Reasons for treatment discontinuation were available for 28 patients. Abbreviations: DFS, disease-free survival; PFS, progression-free survival; OS, overall survival. Footnote: Treatment completion refers to completion of planned adjuvant modified FOLFIRINOX in the resected cohort, whereas ongoing treatment indicates patients who were still receiving first-line FOLFIRINOX at the time of data collection in the metastatic cohort.

**Table 4:** Univariable Cox Proportional Hazards Regression Analysis for Overall Survival in the Resected Cohort.

Variable	HR (95% CI)	*p* Value
Sex	0.652 (0.340–1.250)	0.198
Adjuvant treatment completion	0.576 (0.293–1.133)	0.110
Baseline SMI	0.975 (0.936–1.016)	0.222
Low baseline SMI	1.018 (0.537–1.929)	0.957
Baseline VAT > 100 cm^2^	0.701 (0.360–1.364)	0.295
Follow-up SMI	0.968 (0.923–1.015)	0.178
Low follow-up SMI	0.780 (0.404–1.509)	0.461
Follow-up VAT > 100 cm^2^	0.440 (0.212–0.916)	0.028
ΔSMI (%)	0.996 (0.969–1.024)	0.764
ΔVATI (%)	0.993 (0.984–1.001)	0.092
ΔSATI (%)	0.993 (0.983–1.003)	0.162

**Table 5:** Distribution of Metastatic Disease in the Metastatic Cohort.

Variable	n (%)
Number of metastatic sites †	
1	6 (17.6)
2	7 (20.6)
≥3	21 (61.8)
Metastatic sites ‡	
Liver	42 (82.4)
Lymph node	33 (64.7)
Peritoneum	8 (15.7)
Lung	7 (13.7)
Bone	1 (2.0)

† Data on the number of metastatic sites were available for 34 of 51 patients (66.7%); data were missing for 17 patients (33.3%). Percentages for the number of metastatic sites were calculated using the 34 patients with available data as the denominator. ‡ Patients could have more than one metastatic site; therefore, percentages for individual metastatic sites do not sum to 100%.

**Table 6:** Univariable Cox Proportional Hazards Regression Analysis for Overall Survival in the Metastatic Cohort.

Variable	HR (95% CI)	*p* Value
Sex	0.751 (0.309–1.825)	0.527
CA19-9	1.000 (1.000–1.000)	0.074
Lung metastasis	3.174 (1.347–7.481)	0.008
Baseline SMI	1.000 (1.000–1.000)	0.829
Low baseline SMI	1.256 (0.645–2.445)	0.502
Baseline VAT > 100 cm^2^	1.367 (0.730–2.562)	0.329
Follow-up SMI	0.978 (0.931–1.028)	0.377
Low follow-up SMI	1.140 (0.473–2.746)	0.770
Follow-up VAT > 100 cm^2^	0.655 (0.330–1.299)	0.226
ΔSMI (%)	0.961 (0.929–0.994)	0.022
ΔVATI (%)	0.989 (0.977–1.002)	0.109
ΔSATI (%)	0.993 (0.982–1.003)	0.172

**Table 7:** Multivariable Cox Proportional Hazards Regression Analysis for Overall Survival.

Cohort	Variable	HR (95% CI)	*p* Value
Resected	Follow-up VAT > 100 cm^2^	0.460 (0.220–0.960)	0.039
	Sex	0.670 (0.340–1.310)	0.240
	Adjuvant treatment completion	1.410 (0.690–2.850)	0.347
Metastatic	Lung metastasis	5.792 (1.880–17.841)	0.002
	ΔSMI (%)	0.949 (0.913–0.986)	0.008
	CA19-9	1.000 (1.000–1.000)	0.123
	Sex	0.751 (0.309–1.825)	0.527

The multivariable Cox model for the resected cohort included 47 patients with 38 deaths, whereas the metastatic cohort model included 41 patients with 35 deaths; 10 patients in the metastatic cohort were excluded from the model because of missing covariate data.

## Data Availability

The data presented in this study are available from the corresponding author upon reasonable request. The data are not publicly available due to privacy and ethical restrictions.

## Supplementary Materials

The following supporting information can be downloaded at: https://www.mdpi.com/article/10.3390/jcm15166282/s1; Table S1: STROBE Statement—Checklist of items that should be included in reports of cohort studies.
