# Supplementary material for: Stage-Specific Prognostic Impact of Longitudinal Body Composition Changes in Patients with Pancreatic Ductal Adenocarcinoma Treated with FOLFIRINOX: A Dual-Cohort Study
Source: J Clin Med. 2026 Aug 13;15(16):6282. doi: 10.3390/jcm15166282 (PMC13513821; doi:10.3390/jcm15166282)
Supplement: Supplementary file 1 [file jcm-15-06282-s001.zip › jcm-4461773-supplementary.pdf]

Table S1. STROBE Statement—Checklist of Items That Should Be Included in Reports of Cohort Studies

Manuscript: Stage-Specific Prognostic Impact of Longitudinal Body Composition Changes in Patients with Pancreatic Ductal Adenocarcinoma Treated with FOLFIRINOX: A Dual-Cohort Study

Section/Topic	Item No.	STROBE recommendation	Reported in the manuscript
Title and abstract	1	Indicate the study's design with a commonly used term in the title or abstract; provide an informative and balanced summary of what was done and found.	Title; Abstract (Background, Methods, Results, Conclusions)
Background/rationale	2	Explain the scientific background and rationale for the investigation being reported.	Introduction, paragraphs 1–5
Objectives	3	State specific objectives, including any prespecified hypotheses.	Introduction, final paragraph
Study design	4	Present key elements of study design early in the paper.	Materials and Methods—Study Design and Patient Population
Setting	5	Describe the setting, locations, and relevant dates, including periods of recruitment, exposure, follow-up, and data collection.	Materials and Methods—Study Design and Patient Population; CT Acquisition and Body Composition Analysis
Participants	6(a)	Give eligibility criteria, and the sources and methods of selection of participants. Describe methods of follow-up.	Materials and Methods—Study Design and Patient Population
Participants	6(b)	For matched studies, give matching criteria and number of exposed and unexposed.	Not applicable; this was not a matched cohort study.
Variables	7	Clearly define all outcomes, exposures, predictors, potential confounders, and effect modifiers. Give diagnostic criteria, if applicable.	Materials and Methods—Definitions; Study Outcomes; Statistical Analysis
Data sources/measurement	8	For each variable of interest, give sources of data and details of methods of assessment. Describe comparability of assessment methods if there is more than one group.	Materials and Methods—Treatment; CT Acquisition and Body Composition Analysis; Definitions
Bias	9	Describe any efforts to address potential sources of bias.	Methods—Statistical Analysis (complete-case approach; landmark sensitivity analysis); Discussion—Limitations
Study size	10	Explain how the study size was arrived at.	Materials and Methods—Statistical Analysis: no formal sample-size calculation; all consecutive eligible patients were included.
Quantitative variables	11	Explain how quantitative variables were handled in the analyses. If applicable, describe which groupings were chosen and why.	Materials and Methods—Definitions and Statistical Analysis; VAT >100 cm² and sex-specific low-SMI thresholds described.
Statistical methods	12(a)	Describe all statistical methods, including those used to control for confounding.	Materials and Methods—Statistical Analysis
Statistical methods	12(b)	Describe any methods used to examine subgroups and interactions.	Stage-specific analyses were performed separately for the resected and metastatic cohorts; no formal interaction analysis was reported.
Statistical methods	12(c)	Explain how missing data were addressed.	Materials and Methods—Statistical Analysis; complete-case approach, no imputation.
Statistical methods	12(d)	If applicable, explain how loss to follow-up was addressed.	Study Outcomes and Statistical Analysis; survival outcomes censored at last follow-up. Eligibility required evaluable follow-up CT for longitudinal analyses.
Statistical methods	12(e)	Describe any sensitivity analyses.	Materials and Methods—Statistical Analysis; Results—landmark sensitivity analysis using follow-up CT date as time zero.
Participants	13(a)	Report numbers of individuals at each stage of study—potentially eligible, examined for eligibility, confirmed eligible, included, completing follow-up, and analysed.	Methods—Study Design and Patient Population; Results—Patient Characteristics. Final cohorts: 47 resected and 51 metastatic. Complete historical screening flow could not be reconstructed.
Participants	13(b)	Give reasons for non-participation at each stage.	Methods—Study Design and Patient Population; exact numbers by exclusion reason were not systematically recorded and could not be reconstructed.
Participants	13(c)	Consider use of a flow diagram.	A complete flow diagram could not be generated because historical screening/exclusion counts were unrecoverable; this is explicitly acknowledged in Methods and Discussion.
Descriptive data	14(a)	Give characteristics of study participants and information on exposures and potential confounders.	Results—Patient Characteristics; Table 1; Table 2; Table 3; Table 5
Descriptive data	14(b)	Indicate number of participants with missing data for each variable of interest.	Table 2 footnotes; Table 5 footnote; Table 7 note; Statistical Analysis.
Descriptive data	14(c)	Summarise follow-up time.	CT Acquisition and Body Composition Analysis reports median baseline-to-follow-up CT intervals; survival follow-up is represented through time-to-event analyses.
Outcome data	15	Report numbers of outcome events or summary measures over time.	Results—Treatment Characteristics and Survival Outcomes; Table 3; Table 7 note reports deaths per multivariable model (38

Section/Topic	Item No.	STROBE recommendation	Reported in the manuscript
			resected; 35 metastatic).
Main results	16(a)	Give unadjusted and, if applicable, confounder-adjusted estimates and their precision. Make clear which confounders were adjusted for and why.	Results—Tables 4, 6, and 7; accompanying text; HRs with 95% CIs and p values.
Main results	16(b)	Report category boundaries when continuous variables were categorized.	Materials and Methods—Definitions; low-SMI sex-specific cutoffs and VAT >100 cm^2 threshold.
Main results	16(c)	If relevant, consider translating estimates of relative risk into absolute risk for a meaningful time period.	Not applicable/not performed; primary effect measures were hazard ratios from time-to-event models.
Other analyses	17	Report other analyses done—e.g., subgroup and sensitivity analyses.	Results—separate resected/metastatic cohort analyses; landmark sensitivity analysis.
Key results	18	Summarise key results with reference to study objectives.	Discussion, opening paragraph; Summary of the Main Findings.
Limitations	19	Discuss limitations, taking into account sources of potential bias or imprecision; discuss direction and magnitude of potential bias.	Discussion—Limitations: retrospective single-center design, unreconstructable screening flow, selection/survivorship bias, missing data, modest sample size, residual confounding, treatment-data limitations.
Interpretation	20	Give a cautious overall interpretation considering objectives, limitations, multiplicity of analyses, results from similar studies, and other relevant evidence.	Discussion; conclusions are explicitly framed as exploratory/hypothesis-generating and requiring prospective validation.
Generalisability	21	Discuss the generalisability (external validity) of the study results.	Discussion—Limitations and concluding paragraphs; single-center design and limited generalisability acknowledged.
Funding	22	Give the source of funding and the role of funders.	Funding: This research received no external funding.

Note for submission: The checklist is completed using manuscript sections/tables because the supplied manuscript version is not page-numbered. If the final MDPI Word/PDF proof has stable page numbers, the last column can be updated with page numbers before upload.
